# Assessing different oil sources efficacy in reducing environmental heat-stress effects via improving performance, digestive enzymes, antioxidant status, and meat quality

**DOI:** 10.1038/s41598-023-47356-6

**Published:** 2023-11-17

**Authors:** Ahmed M. Elbaz, Engy F. Zaki, Atif A. Salama, Faisal B. Badri, Hany A. Thabet

**Affiliations:** 1 Animal and Poultry Production Department, Desert Research Center, Cairo, Egypt; 2 Poultry Production Department, Faculty of Agriculture, Ain Shams University, Cairo, Egypt

**Keywords:** Animal behaviour, Animal physiology

## Abstract

Adding oil to the feed of genetically improved broilers is necessary to provide energy requirements, in addition to enhancing metabolism, growth performance, immune response. This study aims to reveal the effect of adding different oil sources in the diets of broilers exposed to environmental heat stress on performance, digestibility, oxidative status, plasma lipids, fatty acids content, and meat quality. Six hundred twenty-five one-day-old broiler chicks were randomly distributed to five groups as follows: the first group fed a diet without oil (CON) as a control, while the second to the fifth group fed a diet containing soy oil (SO), corn oil (CO), olive oil (OO), and fish oil (FO), respectively. Results indicated a significant deterioration in growth performance, carcass traits, and oxidative state with a significant decrease in carcass quality in heat-stressed chickens fed the CON diet. Results showed increased growth, enhanced feed conversion ratio, and carcass dressing in broilers fed the oil-supplemented diet compared to the control diet, however, the digestive enzymes activity was not affected by receiving an oil-supplemented diet. The best performance was in chickens fed OO and SO, compared with FO and CO. Plasma aspartate aminotransferase (AST), and alanine aminotransferase (ALT) increased in broilers fed an oil-supplemented diet. Plasma high-density lipoprotein (HDL), and superoxide dismutase (SOD) remarkably increased in broilers fed OO, whereas the malondialdehyde (MDA) decreased compared to the other groups. Adding different dietary oil sources enhanced the breast muscle's fatty acid composition. Broiler diets supplemented with oils positively affected meat quality by enhancing color measurements, and TBA values, while the best were in chicken fed OO. It was concluded that adding dietary oil at 3% in the diets of broiler chicken exposed to environmental heat stress positively affected growth performance, enhanced oxidative status, and meat quality, best results were in broilers fed a diet that included olive oil.

## Introduction

Understanding the environmental conditions surrounding the bird and how to control it are some of the poultry industry success factors. One of the various environmental stress factors is heat stress, which has a detrimental effect on the health of the bird and its productivity ^1^. Thermal stress results from the relationship between the amount of energy produced by the bird and the amount of energy emitted from the bird's body to the surrounding environment, which causes a defect in the bird's body. Modern broiler breeds have a high metabolic activity that produces a high amount of heat from the body, which makes them more sensitive to heat stress ^2^. The broilers exposure to high temperature leads to behavioral, immune, and physiological changes in an attempt to resist heat stress, which results in significant economic losses in the poultry industry. Experts began to work on solving the problem of heat stress in the poultry industry by developing houses (including building construction, pad cooling systems, and ventilation), however, this development did not give the desired results, as well as the high costs of this technology hindered its use in developing countries. The initial symptom of heat stress exposure is a reduction in feed intake, leading to a deficiency in the necessary nutrients, which compromises the bird's welfare and performance, in addition to increasing mortality^1^. The reduction in feed intake is an attempt to generate less heat to counter the heat stress effect. This made the nutritionists manipulate the diet, whether by changing the composition of the diet or using some additives (e.g., vitamins, minerals, probiotics, and oil) in an attempt to mitigate the harmful effect of heat stress on the bird^3, 4^. Using feed additives led to enhancing nutrient digestibility, immune response, and antioxidant status, as well as, improving gut health (modifying microbial content and histological), thus improving broiler performance under heat stress^5^. The required energy under the conditions of heat stress is higher than it is at the temperature of the zone of thermal neutrality because the additional calories are needed to dissipate body heat. Nutritionists indicated that carbohydrates have a higher heat increment (amount of energy lost as a result of the physical and chemical procedures involved in digestion and metabolism) than fats, therefore a part of the carbohydrates in broiler chicken diets is substituted with fats through the heat stress period^6^. The inclusion of vegetable oils is common in most poultry feed formulations. The energetic value of oils depends in broiler diets on the length of the carbonic chain, the number of double bonds, the composition of the free fatty acid, and the specific arrangements of (saturated and unsaturated) fatty acids. In addition to the fact that oils are a major source of energy, it has an important role in enhancing the absorption of fat-soluble vitamins,^6^ increasing the efficiency of utilization of the consumed energy, and diet palatability^7, 8^. Moreover, adding oil to the diet reduces the rate of food passage in the gastrointestinal tract, which improves the absorption of nutrients in the diet^7^. There is ample evidence confirming that adding fat to poultry feed contributes to improving the bird's growth, performance, immunity, and survivability^9, 10^.

Birds are unable to synthesize many fatty acids; yet, some fatty acids are necessary for metabolism, digestion, and the formation of fatty acids in meat. Many studies have confirmed the relation between fat deposition in the body and the content of fatty acids in the diet^6, 8^. Vegetable oils are rich in digestible unsaturated fatty acids compared to animal fats which are rich in saturated fatty acids^11^. Dietary oil/fat sources impact immunocompetence via many mechanisms; including affecting fatty acid membrane composition, as well as, affecting the other cell-signaling pathways and inflammatory process^12^. Furthermore, some fatty acids are effective in reducing the cecal count of Campylobacter in broilers^13^. Fatty acids, especially omega-6 and omega-3, are proven to be important in maintaining numerous physiological, and biological functions in birds and humans^14^. The ω-6 or ω-3 fatty acids are found in certain foods such as nuts, plants (soybean, olive, corn, and sunflower), and fish oil^15^. Digestion and absorption of fat (nutritional value) are related to many factors, including the fat sources in the diet, which are affected by fatty acid saturation degree, and the condition of the bird. A previous study showed that feeding chickens a diet containing sunflower oil led to a significant decrease in the deposition of abdominal fat compared to animal fat^11^. As another study explained, diets rich in C18:2 had a decrease in the deposition of fat in comparison with diets rich in saturated fatty acids, especially mono-unsaturated fatty acids^16^. The type and quantity of oil added to the feed affect the activity of digestive enzymes, as it has been proven that diets high in saturated fatty acids (SFAs) reduce the secretion of pancreatic lipase, which is the enzyme responsible for digesting fats. On the other hand, diets high in polyunsaturated fatty acids (PUFAs) increase the production and secretion of pancreatic lipase^17^. The use of soybean oil in the broiler diet resulted in better FCR and greater weight gain than those containing tallow and poultry fat^18^. Therefore, we need future studies on the effect of different oil sources in terms of the composition of fatty acids, the positional distribution of fatty acids (on the glycerol molecule), and biologically active compounds on bird performance, and immune function,^19^ and the health of the consumer and the bird.

Increasing interest recently in manipulating the diet, via the use of feed additives or change composition diets, during heat stress in an attempt to mitigate the harmful effects of heat stress on the bird. It has been suggested to use some vegetable oils that are rich in unsaturated fatty acids in addition to their high content of natural antioxidants has been suggested as an economical and effective means of controlling lipid peroxidation during heat stress, as well as, due to owning health benefits and enhancing immune functions and stability to poultry meat, as well as, providing needed energy and better feed efficiency during heat stress. Even though a decent amount of information is available on the effect of using oils on performance index and blood lipid profile^20^, the studies on the impact of different oil sources on some enzymes, immune-oxidative status, metabolic profiles, and meat quality in broilers under heat stress are limited. Hence, our objective was to study the effect of adding different oil resources on the productive performance, digestive enzymes, carcass traits, oxidative status, and meat quality of broilers exposed to environmental heat stress.

## Results

### Productive performance

The addition of different oils in the diets of broilers exposed to environmental heat stress had a positive impact on performance, as shown in Table 1. No differences in BWG and FI during the period from 1 to 21 days among the experimental groups (*p* < 0.05), however, there was an inconsiderable improvement in the BWG of the chicken fed OO and SO compared to other groups, while there was a slight improvement in the FCR with the addition of oil to the diet (*p* < 0.05). FCR was better in birds fed SO and OO (*p* < 0.05) compared to CON, FO, and CO during days 1–21. BWG significantly increased in the broilers fed an oil-included diet (SO, CO, OO, and FO groups), compared to the control during 22–35 d and 1–35 d. BWG increased in chickens fed OO, SO, and CO (*p* < 0.05) compared to CON and FO during d 22–35. FCR significantly decreased in chickens fed an oil included diet at 35 d. FO, CO, and SO groups had better FCR than CON, but worse than OO at d22-35, however, there was a significant similarity among SO and CO groups. Chickens fed OO, SO, and CO had better performance (BWG and FCR) than the control group (*p* < 0.05), only the OO group was better. In addition, the EPEF of the groups fed an OO and SO diet was significantly higher (*p* < 0.05) at 35 d compared to the CO, FO, and the control, however, OO was better.

**Table 1:** Effect of adding different dietary oil sources on growth performance of broilers under environmental heat stress

Items	CON	SO	CO	OO	FO	SEM	p-Value
1–21 day							
BWG (g)	761	796	779	803	773	17.04	0.151
FI (g)	1042	1037	1051	1040	1035	33.70	0.288
FCR	1.37^a^	1.30^b^	1.35^a^	1.29^b^	1.33^ab^	0.035	0.024
22–35 day							
BWG (g)	882^b^	969^a^	955^a^	978^a^	926a^b^	21.66	0.045
FI (g)	2011	2060	2006	2026	2038	31.04	0.164
FCR	2.278^a^	2.126^b^	2.101^b^	2.069^c^	2.207^b^	0.108	0.011
1–35day							
BWG (g)	1642^d^	1765^b^	1736^b^	1780^a^	1698^c^	19.06	< 0.001
FI (g)	3051	3097	3058	3063	3074	37.45	0.173
FCR	1.827^a^	1.757^b^	1.762^b^	1.719^c^	1.809^ab^	0.081	< 0.001
EPEF	263^b^	275^ab^	268^b^	284^a^	265^b^	9.115	0.027

^a–c^Means within the same row with different superscripts differ. *CON* without additional oil, *SO* soy oil, *CO* corn oil, *OO* olive oil, *FO* fish oil, *SEM* standard error of means. *BWG* body weight gain, *FI* feed intake, *FCR* feed conversion ratio (g/g), *EPEF* European Production Efficiency Factor.

### Carcass characteristics

The carcass characteristics of the broiler were summarized as shown in Table 2. Breast, thigh, and liver percentages were not significantly affected by the experimental groups. On the contrary, there was a significant difference in dressing and abdominal fat between the experimental treatments. Dietary oils enhanced the percentage of dressing of broilers exposed to environmental heat stress compared to the control group. Our data showed that the dressing percentage increased in SO, CO, and OO (*p* < 0.05) groups more than those in FO and CON groups. Nevertheless, broilers fed a diet containing oil had lower abdominal fat compared to the control group. Abdominal fat slightly decreased in chickens fed OO and CO (*p* < 0.05) compared to chickens fed SO and FO.

**Table 2:** Effect of adding different dietary oil sources on carcass characteristics and nutrient digestibility of broilers under environmental heat stress.

Items	CON	SO	CO	OO	FO	SEM	P-value
Carcass characteristics							
Dressing (%)	68.21^b^	72.16^a^	71.74^a^	72.37^a^	69.80^b^	0.127	0.031
Breast	24.07	23.94	24.12	24.37	23.89	3.371	0.197
Thigh	16.25	16.17	16.33	16.09	16.31	0.038	0.215
Liver	1.97	1.88	1.95	1.92	2.01	0.022	0.107
Abdominal fat	2.65^a^	1.90^b^	1.78^b^	1.80^b^	1.84^b^	0.007	0.012
Nutrient digestibility							
Dry matter	70.84^b^	72.25^ab^	71.96^ab^	73.18^a^	71.76^ab^	0.387	0.030
Crude Fat	75.60	77.18	76.54	76.28	75.98	0.351	0.067
Crude protein	70.05	71.22	70.68	70.97	71.06	0.215	0.371

^a–c^Means within the same row with different superscripts differ. *CON* without additional oil, *SO* soy oil, *CO* corn oil, *OO* olive oil, *FO* fish oil, *SEM* standard error of means.

### Nutrient digestibility

No significant differences were noticed among the different dietary oil sources in the average values of crude fat (CF), and crude protein (CF) digestibility, as presented in Table 2. Data indicated that including oils in the diet led to increasing dry matter (DM) digestibility, OO and SO groups were better than other experimental groups (*p* < 0.05), however, the best was in chicken fed on OO. Nevertheless, there was a non-significant increase in fat digestibility in chickens fed SO, CO, and OO (*p* = 0.067) compared with FO and CON groups.

### Plasma parameter

All the added oil sources increased plasma HDL values, whereas there were variations in ALT and AST levels according to the added oil, as shown in Table 3. ALT and AST levels significantly decreased in broilers fed the OO and CO (*p* < 0.05) compared to FO, SO, and CON groups. However, the ALT and AST levels were similar in chickens that received CON and SO (*p* < 0.05), while FO was the highest. The plasma content of thyroid hormones, including T3 (triiodothyronine) and T4 (thyroxine) was significantly higher in chicks fed on an oil diet compared to the control group (*p* < 0.05). Compared to the control group, the group that was fed CO had similar T3 levels, while the chickens fed OO, SO, and FO had higher T3 levels (*p* < 0.05). However, T4 levels were not affected among chickens fed the experimental dietary (*p* < 0.05). In addition, no significant differences were recorded among experimental groups regarding the plasma content of lipid profile, including CHO, TRI, and LDL values (*p* < 0.05).

**Table 3:** Effect of adding different dietary oil sources on plasma lipid, liver enzymes, and thyroid hormones of broilers under environmental heat stress.

Items	CON	SO	CO	OO	FO	SEM	P-value
CHO (mg dl^−1^)	238.4	241.7	245.0	243.8	242.9	2.935	0.188
TRI (mg dl^−1^)	255.1	262.1	259.9	257.7	260.5	3.522	0.151
HDL (mg dl^−1^)	43.02^c^	51.52^b^	54.26^b^	59.08^a^	53.45^b^	1.715	0.001
LDL (mg dl^−1^)	98.44	87.51	94.35	89.70	96.11	2.154	0.092
ALT (IUL^−1^)	37.70^ab^	36.21^ab^	33.52^b^	30.85^c^	40.78^a^	2.350	0.027
AST (IUL^−1^)	70.41^ab^	69.44^ab^	67.01^b^	66.19^b^	74.09^a^	4.057	0.020
T4 (nmol L^−1^)	17.2^b^	21.7^a^	21.9^a^	21.6^a^	22.1^a^	1.244	0.031
T3 (nmol L^−1^)	3.25^b^	4.26^a^	3.72^ab^	4.15^a^	4.20^a^	0.975	0.010

^a–c^Means within the same row with different superscripts differ. *CON* without additional oil, *SO* soy oil, *CO* corn oil, *OO* olive oil, *FO* fish oil, *T3* triiodothyronine, *T4* thyroxine, *CHO* cholesterol, *TRI* triglyceride, *HDL* high-density cholesterol, *LDL* low-density cholesterol, *ALT* alanine aminotransferase, *AST* aspartate aminotransferase.

### Digestive and antioxidative enzymes activity

Data in Table 4 displays the impact of oil addition on the activities of digestive enzymes and antioxidative enzymes. Supplemented oils enhanced the oxidation status of broilers exposed to environmental heat stress, where the SOD level increased and the MDA level decreased (*p* < 0.05) while the GPx level was not affected. MDA levels decreased in the chickens fed OO and SO compared to chickens fed CO, and FO. SOD levels increased in chickens fed OO, FO, and SO compared to chickens fed CO, and CON. Furthermore, the digestive enzymes activities (like amylase, trypsin, and lipase) of broilers had no significant differences among the experimental groups (*p* < 0.05). However, there was an insignificant numerical increase in amylase level with the inclusion of OO and CO (*p* = 0.061). In addition, the inclusion of OO, CO, and SO led to an insignificant numerical increase in lipase (*p* = 0.058).

**Table 4:** Effect of adding different dietary oil sources on digestive enzymes activity and antioxidative enzymes of broilers under environmental heat stress.

Items	CON	SO	CO	OO	FO	SEM	P-value
Digestive enzymes							
Amylase (U/ml)	4.07	3.97	4.26	4.38	4.05	2.319	0.061
Trypsin (U/ml)	24.6	25.7	23.9	24.9	25.1	3.527	0.204
Lipase (U/ml)	10.5	11.3	11.6	12.1	10.8	1.703	0.058
Antioxidative enzymes							
MDA (nmol.ml^-1^)	1.845^a^	1.047^c^	1.289^b^	0.812^d^	1.375^b^	0.541	< 0.001
SOD (U.ml^-1^)	101.2^c^	110.4^b^	105.1^c^	128.6^a^	118.4^b^	1.275	< 0.001
GPx (U.ml^-1^)	32.56	37.10	32.41	35.28	33.90	1.556	0.074

^a–c^Means within the same row with different superscripts differ. *CON* without additional oil, *SO* soy oil, *CO* corn oil, *OO* olive oil, *FO* fish oil, *SOD* superoxide dismutase, *MDA* malondialdehyde, *GPx* glutathione peroxidase.

### Fatty acid profiles of breast meat

Data on the fatty acid profile of broiler chicken meat fed different types of oils under heat stress conditions are shown in Table 5. The saturated fatty acids content (SFA) of broiler meat, especially the palmitic acid (C16:0) was not significantly affected by the oil supplements in broiler diets. However, myristic acid (C14:0) increased in the meat of the CON group, while it decreased in the SO group. Oleic acid (C18:1 ω9) content was higher in groups fed on OO and FO compared to other groups. Furthermore, linoleic acid (C18:2 ω6) content was higher in the group fed on SO compared to other groups. Broilers fed FO and OO had significantly higher C22:6 ω3 and C18:1 ω9, respectively, compared with those fed SO and CO. Broilers fed on SO had the highest linolenic acid (C18:3 ω3) while the lowest value was found in the CON group. Compared to the control group, the chickens fed SO and OO showed a significant increase in USFA. Data PUFA: SFA ratio indicated that broilers fed on FO exhibited a lower ratio of PUFA: SFA while broilers fed on SO had a higher ratio than other oil-feeding groups. Data of ω6:ω3 ratio revealed that the meat of broiler fed on fish oil (FO) had the lowest ratio, followed by broiler fed on olive oil (OO) compared to other groups.

**Table 5:** Effect of adding different dietary oil sources on fatty acids content in the breast of broilers under environmental heat stress.

Items	CON	SO	CO	OO	FO	SEM	P-value
C12:0	0.00	0.01	0.02	0.04	0.01	0.009	0.152
C14:0	0.84^a^	0.54^c^	0.71^b^	0.67^b^	0.73^b^	0.051	0.022
C16:0	23.5	22.4	21.6	22.8	23.1	0.134	0.056
C18:0	6.14	6.31	6.28	6.05	6.23	0.075	0.217
C20:0	0.24	0.27	0.22	0.25	0.27	0.211	0.306
C16:1 ω7	4.19^a^	4.25^a^	4.09^a^	3.59^b^	3.76^b^	0.370	0.015
C18:1 ω9	31.8^c^	32.6^b^	32.9^b^	35.1^a^	34.3^a^	0.545	0.033
C18:2 ω6	22.6^b^	24.7^a^	21.8^b^	22.4^b^	21.5^b^	0.412	0.027
C18:3 ω3	0.90^c^	1.50^a^	1.26^b^	1.04^cb^	1.19^b^	0.112	< 0.001
C20:4 ω6	0.07	0.07	0.08	0.08	0.07	0.270	0.721
C22:6 ω3	0.46^d^	0.65^c^	0.69^c^	0.81^b^	1.07^a^	0.185	0.001
∑ SFA	30.71	29.53	28.83	29.81	30.34	0.463	0.183
∑ USFA	61.02^b^	63.73^a^	60.82^b^	63.06^a^	61.91^b^	0.218	0.001
∑ PUFA	24.04^b^	26.92^a^	23.82^b^	24.36^b^	23.82^b^	0.350	0.014
UFA: SFA	1.986^b^	2.157^a^	2.110^a^	2.117^a^	2.041^b^	0.066	0.001
PUFA: SFA	0.777^c^	0.931^a^	0.824^b^	0.813^b^	0.768^c^	0.098	0.006
ω6:ω3 ratio	16.67^a^	11.53^c^	11.21^c^	12.17^b^	9.55^d^	0.574	< 0.001

^a–c^Means within the same row with different superscripts differ. *CON* without additional oil, *SO* soy oil, *CO* corn oil, *OO* olive oil, *FO* fish oil, *SFA* saturated fatty acids, *USFA* unsaturated fatty acids, *PUFA* polyunsaturated fatty acids.

### Physicochemical characteristics of meat

Table 6 shows the physicochemical characteristics of broiler chicken meat fed different types of oils under heat stress conditions. No significant differences were found in pH values for cooking loss and chilling loss of broiler meat. Data of shear force values showed that the meat of CON, FO, and OO feeding groups had lower shear force values (more tender) than the other groups. Color measurements of broiler meat showed significant differences in L* values. The meat of the broiler fed on olive oil (OO) had a higher L* value followed by the meat of the control group (CON), while no significant differences were found between the meat of the SO and CO groups. However, the meat of FO had the lowest value. Furthermore, data showed that the meat of the FO and SO feeding groups had the highest a* value, and no significant differences were found between the other groups. Data of b* value showed that meat of FO, OO, and CON groups had the highest b* and the differences were insignificant. Moreover, no significant differences were found between the SO and CO groups which had the lowest b* value. Data on the TBA value of broiler chicken meat fed different types of oils under heat stress conditions are shown in Table 8. The meat of broilers fed on the FO and OO had the lowest TBA value, while, the highest TBA value was found in the meat of the CON group.

**Table 6:** Effect of adding different dietary oil sources on physicochemical characteristics of broilers under environmental heat stress.

Items	CON	SO	CO	OO	FO	SEM	P-value
Physical analysis							
pH	5.80	5.76	5.75	5.70	5.72	0.326	0.105
Cooking loss (%)	35.13	35.37	34.24	36.87	36.46	0.075	0.094
Chilling loss (%)	11.53	12.17	12.93	12.01	11.54	0.406	0.151
Shear force (Kg/_f_)	2.26^d^	6.42^a^	4.52^b^	3.40^c^	2.73^ cd^	0.350	< 0.001
Color measurements							
L*	54.49^ab^	52.44^b^	52.34^b^	56.15^a^	50.54^c^	1.117	< 0.001
a*	9.32^b^	11.06^a^	8.69^b^	8.23^b^	11.38^a^	0.682	0.001
b*	14.67^a^	12.00^b^	12.87^b^	14.78^a^	14.03^a^	0.290	0.016
Chemical analysis							
TBA (mg MDA/kg)	0.0450^a^	0.0441^a^	0.0338^a^	0.0175^b^	0.0169^b^	0.191	0.013

^a–c^Means within the same row with different superscripts differ. *CON* without additional oil, *SO* soy oil, *CO* corn oil, *OO* olive oil, *FO* fish oil, L* lightness, a* the redness, b* the yellowness, *TBA* thiobarbituric acid.

## Discussion

Heat stress is one of the most important problems that the poultry industry suffers from due to its detrimental effect on performance and meat quality, leading to great economic losses. Usually, oils and fats are used in poultry feed on a large scale to provide the bird's energy needs. In addition, many previous studies indicated that adding oils or fats during high environmental temperature reduces harmful effects by improving chicks' growth and enhancing antioxidant status and welfare^1, 21^.

Nutritionists have used many nutritional modifications or additions in an attempt to mitigate the harmful effects of any stress on the bird. Therefore, this study aimed to add different sources of oils to reduce the negative effects of heat stress on the bird. In the current experiment, broilers showed signs of heat stress, such as panting and straightening the wings. The results of the current study indicated a significant deterioration in the growth performance of chickens fed a diet without oil, while there was an improvement in the BWG and FCR of broiler chickens fed diets supplemented with oil under environmental heat stress. Chickens fed on OO and SO had better growth performance than the other groups, while the best performance was in broilers fed olive oil (OO). This is consistent with the results of previous studies, which stated that adding oil enhanced the growth performance of chickens exposed to high temperatures^21, 22^. The improvement in growth performance can be explained by the fact that the addition of oil led to the palatability of diets, the oil enhances the absorption of fat-soluble vitamins, reduces the dustiness of feeds, and decreases the rate of food passage within the digestive system^23, 24^, thus improving the utilization of nutrients, as well as, an appositive impact on humoral immunity, which is reflected in growth performance. In addition, enhanced growth of broilers fed on oil could be due to higher UFA and medium-chain fatty acids (MCFA), MCFA is burned exclusively and rapidly for energy production^25^. In this study, there was a difference in the rate of improvement in BWG and FCR between the different types of oil added, where olive oil had the best BWG and FCR. The improved performance in chickens fed olive oil is either due to its high content of monounsaturated fatty acids (MUSF) compared to saturated fatty acids (SFA) or to the balance between the ratio of omega 3 and omega 6 (1:7), that ratio is essential because elevated ω-6 reduces the benefits of ω-3. Furthermore, it contains a large proportion of omega-9, which makes it one of the best and healthiest oils. Olive oil also contains some biologically active compounds that have a role in reducing the oxidative stress harmful effects during the stress period, which is reflected in the enhancement of broiler health and performance. Our results confirm that chickens fed an oil-included diet had better tolerance to environmental heat stress than chickens fed a ration without oil. The group fed on olive oil had the most tolerance to environmental heat stress than other groups. The reason for the variance in growth performance can be explained by the content of each oil of saturated and unsaturated fatty acids. Similar results were obtained by Nobakht et al.^24^ and Khatun et al.^26^. Moreover, the FI was not affected by the inclusion of the dietary oils, this result was in agreement with Khatun et al.^26^. In contrast, some studies showed that FI was affected by the type of oil added to the diet by Ayed et al.^27^. This inconsistency in the results could be due to the conditions of the experiment in terms of chick strain, the age of chickens, feed composition, or oil type and levels. Fatty acids, especially omega-3, have the potential to lessen oxidative stress, improve immune function, altered lipid synthesis, and reduce inflammation leading to improving gut health, and resulting in improving nutrient transport^28^, thus improving growth performance, this was confirmed by the current study.

One of the biggest losses that occur in the poultry industry is the deterioration in carcass traits during environmental heat stress. Our results indicated that exposure of broilers to environmental heat stress led to impairment of carcass traits in the control group. Our results indicated that adding different dietary oil sources to the diet improved carcass traits by increasing the dressing and reducing the content of abdominal fat. Similar results were obtained by Lu et al.^29^, who found an increase in carcass yield and a decrease in abdominal fat with the addition of oil to the diets. The lower abdominal fat content may be due to the role that the added oil plays in regulating the deposition of fat in the carcass. Some studies showed that the types of fatty acids affect the quality of the carcass through the ease of deposition and oxidation of unsaturated fatty acids^30^. In addition, the carcass weight is a proportional measurement of the live body weight of broilers, the increase in carcass meat was due to a proportional reduction of abdominal fat weight or improvement in BWG. The decrease in abdominal fat content can be explained by the role of dietary oils in promoting fatty acid β-oxidation (by decreasing the number of abdominal adipose cells) and inhibiting the absorption of dietary fat and fatty acid biosynthesis, and thus the abdominal fat percentage decreased significantly in the current study^30^. The decrease in abdominal fat resulting from adding oil to the diet is of great economic importance to the poultry industry, as abdominal fat is considered waste because it reduces carcass yield^31, 32^. Furthermore, the distribution of fat between the different parts of the carcass tissues is of great importance to the quality, flavor, and freshness of the carcass and its nutritional value^33^. Our results confirm the effect of supplementing with oil on regulating the distribution of fat inside the carcass, which has a significant economic role in addition to enhancing meat quality.

Nutrient digestibility was studied to determine the extent to of chicken benefited from nutrients under the conditions of high temperatures. In the current study, the digestion of CF and CP was not affected, while the DM increased with the addition of oil compared to the control group. However, a slight increase in CF was observed in SO, CO, and OO groups compared to FO and CON groups. The effect of dietary oil on the digestion of nutrients varies based on the composition of the oil, the type, and the length of saturated or unsaturated fatty chains^34^. Nevertheless, the reason for the improvement in the digestion of DM can be attributed to the fact that the addition of oil led to a reduction in the rate of feed passage in the gut, which enhances the ability to digest and absorb nutrients^35^. Results of the current study indicated that there was no effect from different dietary oil sources on the activity of digestive enzymes. These results are similar to previous findings^36^. However, there was a numerical increase in amylase activity with the inclusion of OO and CO. In addition, the inclusion of OO, CO, and SO led to a numerical increase in lipase. A previous study indicated higher activities of some enzymes when supplementing with different oil sources^37^. In general, the digestive enzyme activities were consistent with improved growth under hot conditions. It's known that heat stress leads to reduced feed intake, resulting in an imbalance in energy and small intestine functions (impairs nutrient absorption) through the change in the expression of the digestive enzymes (lipase, amylase, trypsin). During the heat stress period, amylase, lipase, and trypsin expression significantly decreased as a result of the impairment in intestinal function and structure^30, 38^. Most importantly, heat stress reduces intestinal expression of the fatty acid-binding protein, which is involved in fatty acid uptake and transport^39^. In addition, a study found certain feed additives that target reducing oxidative stress and inflammation^28^, such as n-3 fatty acids, enhanced gut health in addition to enhancing nutrient absorption in the small intestine. In conclusion, the addition of dietary oils may have a role in changing gene expression, which may increase the digestive enzymes activity, thus enhancing nutrient digestion and absorption.

The plasma biochemistry of birds varied by the different oil sources, and there were noticeable effects on liver enzymes and thyroid hormones. The composition of fatty acids in the diet had a significant effect on the features of plasma lipids, despite that, blood fats were not affected in our study which could be due to the oil burning for energy and dissipation of heat stress that the bird was exposed to^40^. This beneficial effect on the health of the broiler is indicated by the increase in the level of HDL in the plasma. Oil source has a noticeable effect on the concentration of AST and ALT as it decreased in the OO and CO groups. This result can be explained by the beneficial effect of PUFA on the safety of the liver by maintaining the integrity of the liver cell membrane and the fact that some oils contain antioxidants and antimicrobial effects that enhance liver functions^9, 41^. Thyroid hormones were measured for their important role in the metabolism process^42^. Our results show a decrease in the level of thyroid hormones (T3 and T4) in the control group, which is in correspondence to Dahlke et al.^43^ and Sohail et al.^44^, who noticed a decrease in T3 and T4 levels in birds exposed to high temperatures. Plasma levels of T3 and T4 increased as a result of feeding chickens an oil-supplemented diet in this experiment, which led to an improvement in broiler performance.

The oxidation status was evaluated to compare to what the previous studies showed of the harmful effect of oxidative stress during heat stress on the performance of the bird^45, 46^. In the current study, the oxidation state improved significantly by including oils in the diet. This was confirmed by the increase in SOD and the decrease in MDA in plasma. In addition, there were clear, insignificant differences in the extent of improvement in the oxidation state depending on the oil source due to the biologically active compounds in each oil (such as tocopherols). Biological compounds play an important role in protecting the cell membrane from free radicals, which leads to enhancing chicken health^47^. The results of this study confirm the beneficial effect of adding oils to diets on enhancing the oxidative capacity of broilers exposed to environmental heat stress, which contributes to reducing oxidative degradation, resulting in strengthening immune functions and stability of poultry meat during heat stress. This is consistent with the results presented by Rymer et al.^48^ and Lindblom et al.^49^.

Consumers are becoming interested in the importance of keeping track of the health aspects associated with food. Since poultry is the main source of protein in developing countries because it contains a low content of fat, which is associated with the prevention of some diseases. As indicated by previous studies that the amount or composition of fatty acids in chicken tissues can be modified by manipulating the composition of dietary fatty acids^50^. Feeding broilers with different oils significantly affected the fatty acid profile of broiler chicken meat. Higher content of oleic acid (C18:1 ω9) was found in broilers fed on olive oil (OO). These results are consistent with the data of Zhang et al.^51^ who indicated that the meat of broilers fed a diet supplemented with different levels of olive oil had the highest content of oleic acid (C18:1 ω9). Long et al.^52^ demonstrated that the addition of fish oil to broiler diets significantly increased the oleic acid content in broiler breast meat. The highest content of linoleic acid and linolenic acid was found in the meat of the broiler fed on soybean oil (SO). These results are consistent with that obtained by Zaki et al.^53^, and Ayed et al.^27^ who indicated that a higher content of linoleic acid and linolenic acid were found in the meat of broiler fed on rations containing soybean oil. The highest PUFA: SFA ratio was found in broilers fed on SO, while the lowest ratio was found in the FO feeding group. These results agree with Zaki et al.^53^ who found that feeding broilers soybean oils had a higher PUFA: SFA ratio than other feeding groups. Furthermore, Long et al.^52^ reported that the addition of fish oil to broiler diets significantly lowered the PUFA: SFA ratio. Types of oil significantly affected the ω6:ω3 ratio. The lowest ratio was found in the FO group. These results are close to that obtained by Panda et al.^54^ who indicated that the incorporation of fish oil in broiler diets resulted in a significant decrease in the ω6:ω3 ratio. Moreover, Ibrahim et al.^55^ found that supplementing broiler diets with fish oils reduced the ω6:ω3 ratio in broiler chicken meat. In the present study, the meat of broilers fed OO, CO, and SO had a higher ratio of UFA: SFA than those fed FO, and control, which indicates that the broiler's consumption of OO, CO, and SO posed a lower risk of diseases such as heart diseases. Our results confirmed that adding oil to a broiler diet modified breast muscle fatty acid profile.

In our results, different dietary oil sources had no significant effect on the pH values of meat. These results are consistent with the data of Ebeid et al.^56^ and Pekel et al.^57^ who found that different dietary oil sources had no significant effects on the pH value of meat. Data on cooking loss indicated that supplementing broiler diets with different types of oils had no significant effect on the cooking loss of chicken meat. These results are agreeing with Tavárez et al.^45^ and Pekel et al.^57^ who found that different sources of oils had no significant effect on cooking loss of breast chicken meat. Our data on chilling loss is consistent with Omojola et al.^58^ who found that supplementing broiler diets with different oil sources had no significant effect on chilling loss. Nevertheless, our results indicated that feeding on different sources of oil significantly affected on shear force value of broiler meat. The results of the current study contradict the findings of Ebeid et al.^56^ who found insignificant differences in the meat of chickens fed on different types of oils. Pekel et al.^57^ indicated that feeding broilers on diets supplemented with different types of oils had no significant effect on the shear force of meat. Data of color measurements indicated that feeding broilers on different dietary oil types significantly affected on color characteristics of broiler chicken meat in our result. This result is similar to Ayed et al.^27^ who found significant differences in color measurements of broiler chicken meat fed on diets supplemented with different oils. In contrast, Pekel et al.^57^ found that feeding broilers on diets supplemented with different oils had no significant effect on meat color.

In our study, feeding broiler chickens with different sources of oils under heat stress conditions had a significant effect on TBA values on broiler meat. Attia et al.^26^ reported that during hot weather conditions, the TBA value increased due to the peroxidation process, which is similar to our results. Supplementing broiler diets with oils significantly affected on oxidative stability of chickens exposed to heat stress^59^. Similar results were obtained by Abdulla^60^ who indicated that feeding broiler chicken with different dietary oils significantly affected the TBA values of chicken meat. The same results were discovered by Ebeid et al.^56^ who stated that feeding broilers with different types of oils significantly improved the antioxidant status of meat. In the current study, the results indicated that the lowest TBA values were in the OO and FO feeding groups. This result is similar to that obtained by Tufarelli et al^61^. who found that supplementing the broiler diet with olive oil significantly affected the TBA value of meat compared to the other dietary oils due to its high content of phenolic compounds which increases the oxidative stability in meat. Similarly, Ibrahim et al.^55^ indicated that supplementing the broiler diet with fish oil significantly decreased the TBA value compared with different types of oils. It can be concluded that the addition of oil to broiler diets, especially olive oil, and fish oil, improved meat quality, which encourage consumers to purchase broiler meat.

## Conclusion

Supplementing with dietary oils has proven to be effective in alleviating the detrimental effects of environmental heat stress in broilers. Adding different dietary oil sources led to an improvement in growth performance, and enhanced the oxidative status, and plasma lipid index, furthermore, it improved carcass characteristics, and meat quality in broilers exposed to environmental heat stress. Olive oil and soybean oil, respectively, had the best effect on performance and meat quality. However, we need further studies to reveal the effect of mixing dietary oil or using individual fatty acids as a means of reducing the harmful effects of heat stress on chicken performance.

## Materials and methods

The Animal Care and Research Ethics Committee of Desert Research Center, Egypt, approved the animal care protocol used for this experiment.

### Birds, diets, and experimental design

The experiment was conducted at the Siwa Research Station of the Desert Research Center. The experiment began in June 2021 (during summer), temperature and humidity were recorded twice daily (12 am and 12 pm), the average temperature was 31.7 ℃ and the relative humidity was 43% during the experimental period. The temperature was maintained the first day at 34 ℃, the second day at 33 ℃, the third and fourth days at 32 ℃, and starting from the fifth day the chicks were exposed to the ambient temperature until the end of the experiment. Six hundred twenty-five broiler chicks (Ross 308, unsexed) were purchased from a commercial hatchery in Cairo—Egypt, and distributed randomly to five groups, each having 5 replicates (25 chicks in each replicate). Chicks of the first group were fed a diet without adding oil, and the second to the fifth group were fed a diet containing 3% soybean oil, corn oil, olive oil, and fish oil, respectively. Chicks were fed at two phases: the first was the starter diet from the age of 1 to 21 days and the second was the grower diet from the age of 22–35 days (Tables 7, 8), the feed compositions were formulated based on the National Research Council^62^. Feed and water were offered ad libitum, and the lighting was continued throughout the trial period.

**Table 7:** Compositions of starter diet (day 1–22).

Ingredients	Control	SO	CO	OO	FO
Corn (grain)	62.90	54.90	54.90	54.90	54.90
Soybean meal	21.54	38.15	38.15	38.15	38.15
Corn Gluten meal	11.50	0.00	0.00	0.00	0.00
Oil	0.00	3.00	3.00	3.00	3.00
Di calcium phosphate	2.10	2.00	2.00	2.00	2.00
Calcium carbonate	1.28	1.30	1.30	1.30	1.30
Hcl-lysine	0.05	0.00	0.00	0.00	0.00
D-methionine	0.13	0.15	0.15	0.15	0.15
Salt	0.25	0.25	0.25	0.25	0.25
Premix*	0.25	0.25	0.25	0.25	0.25
Total	100	100	100	100	100
Chemical composition					
ME (kcal/kg)	3061	3058	3063	3051	3047
CP (%)	23	23	23	23	23
Ca (%)	1.045	1.047	1.047	1.047	1.047
AP (%)	0.504	0.499	0.499	0.499	0.499

*Each 1 kg of premix (vitamins and minerals) contained: Vitamin A, 10 000 IU, Vitamin D3, 1800 UI; Vitamin E, 15 mg; vitamin K3, 4.5 mg; Vitamin B1, 0.5 mg; Vitamin B2, 4 mg; Vitamin B12, 0.001 mg; Folic acid, 0.1 mg; Pantothenic acid,7 mg; Nicotinic acid, 20 mg; I, 1 mg; Mn, 60 mg; Cu, 5.5 mg ,Zn, 75.

mg; Fe, 40 mg; Co, 0.3 mg; Se, 0.1 mg; Robenidine,52.8 mg.

**Table 8:** Compositions of grower diet (day 22–35).

Ingredients	Control	SO	CO	OO	FO
Corn (grain)	67.80	60.30	60.30	60.30	60.30
Soybean meal	17.50	33.10	33.10	33.10	33.10
Corn gluten meal	11.00	0.00	0.00	0.00	0.00
Oil	0.00	3.00	3.00	3.00	3.00
Di calcium phosphate	1.92	1.90	1.90	1.90	1.90
Calcium carbonate	1.03	1.00	1.00	1.00	1.00
Hcl-Lysine	0.15	0.00	0.00	0.00	0.00
D-Methionine	0.10	0.20	0.20	0.20	0.20
Salt	0.25	0.25	0.25	0.25	0.25
Premix*	0.25	0.25	0.25	0.25	0.25
Total	100	100	100	100	100
Chemical composition					
ME (kcal/kg)	3109	3117	3122	3113	3107
CP (%)	21	21	21	21	21
Ca (%)	0.900	0.900	0.900	0.900	0.900
AP (%)	0.462	0.467	0.467	0.467	0.467

*Each 1 kg of premix (vitamins and minerals) contained: Vitamin A, 10 000 IU, Vitamin D3, 1800 UI; Vitamin E, 15 mg; vitamin K3, 4.5 mg; Vitamin B1, 0.5 mg; Vitamin B2, 4 mg; Vitamin B12, 0.001 mg; Folic acid, 0.1 mg; Pantothenic acid,7 mg; Nicotinic acid, 20 mg; I, 1 mg; Mn, 60 mg; Cu, 5.5 mg ,Zn, 75 mg; Fe, 40 mg; Co, 0.3 mg; Se, 0.1 mg; Robenidine,52.8 mg.

### Performance response

Live body weight (LBW), feed intake (FI), and mortality count of each replicate were recorded daily, and the feed conversion ratio (FCR), and the European Production Efficiency Factor (EPEF) were calculated at days 21, and 35. At 35 days of age, six chicks from each treatment group were randomly taken to represent all treatment replicates to measure parameters. The feed was removed 12 h before slaughter and carcass characteristics were estimated. The weight of carcass (dressing), abdominal fat, liver, thigh muscle, and breast muscle were recorded and expressed as a ratio to live body weight.

$${\text{FCR}} = {\text{the}}\;{\text{feed}}\;{\text{intake}}\;{\text{g/body}}\;{\text{weight}}\;{\text{gain }}\left( {\text{g}} \right)\;{\text{ratio}}$$

$${\text{EPEF}} = {{\left( {{\text{Livability}}\;(\% ) \times {\text{BW}}\;\left( {\text{g}} \right)} \right)} \mathord{\left/ {\vphantom {{\left( {{\text{Livability}}\;(\% ) \times {\text{BW}}\;\left( {\text{g}} \right)} \right)} {\left( {{\text{age}}\;\left( {{\text{days}}} \right) \times {\text{FCR}}} \right)}}} \right. \kern-0pt} {\left( {{\text{age}}\;\left( {{\text{days}}} \right) \times {\text{FCR}}} \right)}} \times 100$$

### Nutrient digestibility

At the end of the trial period (35 days), six broilers per group were weighed and individually caged in metabolic pens and starved for 8 h to begin the digestion experiment, and their excreta were collected for three days (three times a day). The approximate analysis of the dry matter, crude fat, and crude protein of diets and dried excreta were estimated according to AOAC^63^.

### Plasma biochemical analysis

Ten blood samples from each group (two samples from each replicate) at day 35 were collected from the brachial vein with a vacationer heparinized tube and separated by centrifugation at 2500×*g* for 15 min at 4 °C to determine the plasma biochemical constituents. Triglyceride, total cholesterol, low-density cholesterol (LDL), and high-density cholesterol (HDL) concentrations in the plasma were estimated. In addition, the activities of plasma alanine aminotransferase (ALT), aspartate aminotransferase (AST), superoxide dismutase (SOD), malondialdehyde (MDA), as well as the glutathione peroxidase (GPx) were determined by using commercial kits (Spinreact Co. Girona, Spain). Plasma thyroxine (T4) and triiodothyronine (T3) hormone concentrations were assayed by radioimmunoassay with a commercial kit, as evidence of thyroid status.

### Digestive enzymes activity

Intestine content samples were collected from different regions (duodenum, jejunum, and ileum), and preserved in a neutral saline solution, then the mixture was centrifuged (1,792 g for 15min), and the supernatant was separated to estimate the concentrations of digestive enzymes. Lipase, amylase, and trypsin activity were determined using the method described by Sklan and Halevy^64^, Pinchasov et al.^65^, and Sklan et al.^66^, respectively.

### Fatty acid profiles

Total lipid was extracted from a 5g breast muscle sample (left side) with chloroform: methanol (2:1, v/v) as described by Folch et al.^67^. Lipids methyl esters were extracted according to the procedure of Peisker^68^, then analyzed by gas chromatography (GA-17A, Shimadzu, Tokyo, Japan) under programmed temperature conditions. The column was heated to 230°C at 1.5°C/min. A carrier gas such as nitrogen was used at a constant flow rate of 1/50. According to the retention time and peak areas of known calibration standards, the peak is determined in breast fatty acid methyl esters.

### Meat quality indicators

Thiobarbituric acid (TBA) in minced breast meat samples was determined according to the method of Pearson^69^. The amounts of TBA values were expressed as mg of malondialdehyde per kg of meat (mg MDA/kg). pH values of raw chicken meat for each treatment were determined according to Hood^70^. By using a digital pH meter (Jenway 3310 conductivity and pH meter). The pH value was calculated by the average of 3 readings of pH for each treatment.

Shear force: Cooked chicken meat samples of each treatment were sheared three times at different positions by using the Intron Universal Testing Machine (Model 2519–105, USA). The average shear force was calculated from the three obtained results (Kg/f). Cooking: Chicken meat samples of each treatment were cooked in a water bath at 85 °C until the internal temperature reached 75 ± 3 °C. The cooking loss was determined as described by Meek et al.^71^. Chilling loss: Thawing loss of chicken meat samples was calculated according to Omojola et al.^58^.

Color measurements: Color measurements of raw chicken meat were measured by using a Chroma meter (Konica Minolta, model CR 410, Japan) calibrated with a white plate and light trap supplied by manufacturer^72^. The color was expressed as L* (lightness), a* (redness), and b* (yellowness). Color measurements were calculated as the average of three spectral readings at different locations for each treatment.

### Statistical analysis

The data were analyzed with the Statistical Analysis System [SAS, 73]^73^, using one-way analysis of variance (ANOVA), and the generalized linear model (GLM) procedure. Significance was evaluated at the level of *p* < 0.05.

### Ethical approval

Recommended experimental procedures were followed to maintain the welfare and safety of animals following international guidelines of the Committee on Animal Rights at the Desert Research Center. (Approval No. 2021–206). All protocols were carried out in accordance with the Universal Directive on the Protection of Animals Used for Scientific Purposes. All protocols follow the ARRIVE guidelines for reporting animal research (https://arriveguidelines.org).

## Data Availability

All data used during the current study are available from the corresponding author upon reasonable request.

## References

[1] Abdel-Moneim AME (2021). Nutritional manipulation to combat heat stress in poultry–A comprehensive review. J. Therm. Biol.

[2] Deeb N, Shlosberg A, Cahaner A (2002). Genotype-by-environment interaction with broiler genotypes differing in growth rate. 4. Association between responses to heat stress and to cold-induced ascites. Poult. Sci..

[3] Elbaz AM, Ibrahim NS, Shehata AM, Mohamed NG, Abdel-Moneim AME (2021). Impact of multi-strain probiotic, citric acid, garlic powder or their combinations on performance, ileal histomorphometry, microbial enumeration and humoral immunity of broiler chickens. Trop. Anim. Health Prod..

[4] Abdel-Moneim AME, Shehata AM, Mohamed NG, Elbaz AM, Ibrahim NS (2021). Synergistic effect of Spirulina platensis and selenium nanoparticles on growth performance, serum metabolites, immune responses, and antioxidant capacity of heat-stressed broiler chickens. Biol. Trace Element Res..

[5] Wasti S, Sah N, Mishra B (2020). Impact of heat stress on poultry health and performances, and potential mitigation strategies. Animals.

[6] Syafwan S, Kwakkel RP, Verstegen MWA (2011). Heat stress and feeding strategies in meat-type chickens. World's Poult. Sci. J..

[7] Cherian G (2015). Nutrition and metabolism in poultry: Role of lipids in early diet. J. Anim. Sci. Biotechnol.

[8] Upadhayay UPPDD, Vishwa PCV (2014). Growth promoters and novel feed additives improving poultry production and health, bioactive principles and beneficial applications: the trends and advances—A review. Int. J. Pharmacol.

[9] Poorghasemi M, Seidavi A, Qotbi AAA, Laudadio V, Tufarelli V (2013). Influence of dietary fat source on growth performance responses and carcass traits of broiler chicks. Asian-Australas. J. Animal Sci..

[10] Sadeghi AA, Mohammadi A, Shawrang P, Aminafshar M (2013). Immune responses to dietary inclusion of prebiotic-based mannan-oligosaccharide and β-glucan in broiler chicks challenged with Salmonella enteritidis. Turkish J. Veterin. Animal Sci..

[11] Shoaib M, Bhatti SA, Ashraf S, Hamid MMA, Javed MM, Amir S, Aslam N, Roobi A, Iqbal HH, Asif MA, Nazir U (2023). Fat digestion and metabolism: effect of different fat sources and fat mobilisers in broilers diet on growth performance and physiological parameters—A review. Ann. Animal Sci..

[12] Baião NC, Lara LJC (2005). Oil and fat in broiler nutrition. Brazil. J. Poult. Sci..

[13] Hermans D, Martel A, Garmyn A, Verlinden M, Heyndrickx M, Gantois I, Haesebrouck F, Pasmans F (2012). Application of medium-chain fatty acids in drinking water increases Campylobacter jejuni colonization threshold in broiler chicks. Poult. Sci..

[14] Samman S (2009). Fatty acid composition of certified organic, conventional and omega-3 eggs. Food Chem..

[15] Khatun J, Loh TC, Akit H, Foo HL, Mohamad R (2017). Influence of different sources of oil on performance, meat quality, gut morphology, ileal digestibility and serum lipid profile in broilers. J. Appl. Anim. Res..

[16] Smink W, Gerrits WJJ, Hovenier R, Geelen MJH, Verstegen MWA, Beynen AC (2010). Effect of dietary fat sources on fatty acid deposition and lipid metabolism in broiler chickens. Poult. Sci..

[17] Szabó RT, Kovács-Weber M, Zimborán Á, Kovács L, Erdélyi M (2023). Effects of short-and medium-chain fatty acids on production, meat quality, and microbial attributes—A review. Molecules.

[18] Zhang B, Haitao L, Zhao D, Guo Y, Barri A (2011). Effect of fat type and lysophosphatidylcholine addition to broiler diets on performance, apparent digestibility of fatty acids, and apparent metabolizable energy content. Animal Feed Sci. Technol..

[19] Swiatkiewicz S, Arczewska-Wlosek A, Jozefiak D (2015). The relationship between dietary fat sources and immune response in poultry and pigs: An updated review. Livestock Sci..

[20] Fouad AM, El-Senousey HK (2014). Nutritional factors affecting abdominal fat deposition in poultry: a review. Asian-Australas. J. Animal Sci..

[21] Ghazalah AA, Abd-Elsamee MO, Ali AM (2008). Influence of dietary energy and poultry fat on the response of broiler chicks to heat therm. Int. J. Poult. Sci.

[22] Chwen LT, Foo HL, Thanh NT, Choe DW (2013). Growth performance, plasma Fatty acids, villous height and crypt depth of preweaning piglets fed with medium chain triacylglycerol. Asian-Australas. J. Animal Sci..

[23] Nagadi SA, de Oliveira AA (2019). Dietary distilled fatty acids and antioxidants improve nutrient use and performance of Japanese male quails. Animal Sci. Papers Rep..

[24] Nobakht A, Tabatbaei S, Khodaei S (2011). Effects of different sources and levels of vegetable oils on performance, carcass traits and accumulation of vitamin E in breast meat of broilers. Curr. Res. J. Biol. Sci.

[25] Rubin M (2000). Long-chain fatty acids; in long-term home parenteral nutrition: a double-blind randomized cross-over study. Nutrition.

[26] Khatun J, Loh TC, Akit H, Foo HL, Mohamad R (2018). Influence of different sources of oil on performance, meat quality, gut morphology, ileal digestibility and serum lipid profile in broilers. J. Appl. Animal Res..

[27] Ayed HB, Attia H, Ennouri M (2015). Effect of oil supplemented diet on growth performance and meat quality of broiler chickens. Adv. Techniq. Biol. Med..

[28] Wang J, Clark DL, Jacobi SK, Velleman SG (2021). Supplementation of vitamin E and omega-3 fatty acids during the early posthatch period on intestinal morphology and gene expression differentiation in broilers. Poult. Sci..

[29] Lu Q, Wen J, Zhang H (2007). Effect of chronic heat exposure on fat deposition and meat quality in two genetic types of chicken. Poult. Sci..

[30] Yi D (2016). N-acetylcysteine improves the growth performance and intestinal function in the heat-stressed broilers. Anim. Feed Sci. Technol.

[31] Jennen, D.G. Chicken fatness: From QTL to candidate gene. (2004).

[32] Elbaz AM, Zaki EF, Morsy AS (2022). Productive performance, physiological responses, carcass traits, and meat quality of broiler chickens fed quinoa seeds. Adv. Anim. Vet. Sic.

[33] Wood (2008). Fat deposition, fatty acid composition and meat quality: A review. Meat Sci..

[34] Jansen M, Nuyens F, Buyse J, Leleu S, Van Campenhout L (2015). Interaction between fat type and lysolecithin supplementation in broiler feeds. Poult. Sci..

[35] Aardsma MP, Mitchell RD, Parsons CM (2017). Relative metabolizable energy values for fats and oils in young broilers and adult roosters. Poult. Sci..

[36] Attia YA, Al-Harthi MA, Abo El-Maaty HM (2020). The effects of different oil sources on performance, digestive enzymes, carcass traits, biochemical, immunological, antioxidant, and morphometric responses of broiler chicks. Front. Vet. Sci..

[37] Jang IS, Ko YH, Kang SY, Lee CY (2007). Effect of a commercial essential oil on growth performance, digestive enzyme activity and intestinal microflora population in broiler chickens. Animal Feed Sci. Technol..

[38] He X (2018). Chronic heat stress damages small intestinal epithelium associated with ampk pathway in broilers. J. Agric. Food Chem.

[39] Habashy WS (2017). Effect of heat stress on protein utilization and nutrient transporters in meat-type chickens. Int. J. Biometeorol.

[40] Wang J, Zhu Q, Ahmad H, Zhang X, Wang T (2013). Combination of linseed and palm oils is a better alternative than single oil for broilers exposed to high environmental temperature. J. Poult. Sci..

[41] Bhatnagar AS, Prasanth Kumar PK, Hemavathy J, Gopala Krishna AG (2009). Fatty acid composition, oxidative stability, and radical scavenging activity of vegetable oil blends with coconut oil. J. Am. Oil Chem. Soc..

[42] Marietto-Gonçalves GA, Martins TF, Almeida SM, Lima ET, Andreatti-Filho RL (2006). Presença de cistos de Balantidium sp. em amostras fecais aviárias. Nosso Clín.

[43] Dahlke F (2005). Avaliação de diferentes fontes e níveis de selênio para frangos de corte em diferentes temperaturas. Arch. Vet. Sci..

[44] Sohail MU (2010). Alleviation of cyclic heat stress in broilers by dietary supplementation of mannan-oligosaccharide and Lactobacillus-based probiotic: Dynamics of cortisol, thyroid hormones, cholesterol, C-reactive protein, and humoral immunity. Poult. Sci..

[45] Tavárez MA (2011). Effect of antioxidant inclusion and oil quality on broiler performance, meat quality, and lipid oxidation. Poult. Sci..

[46] Boler DD (2012). Effects of oxidized corn oil and a synthetic antioxidant blend on performance, oxidative status of tissues, and fresh meat quality in finishing barrows. J. Animal Sci..

[47] Attia, Y. A., Böhmer, B. M., & Roth-Maier, D. A. Responses of broiler chicks raised under constant relatively high am-bient temperature to enzymes, amino acid supplementations, or a high-nutrient diet. *Arch. für Geflugelkd*, **70** (2006).

[48] Rymer C, Gibbs RA, Givens DI (2010). Comparison of algal and fish sources on the oxidative stability of poultry meat and its enrichment with omega-3 polyunsaturated fatty acids. Poult. Sci..

[49] Lindblom SC, Gabler NK, Bobeck EA, Kerr BJ (2019). Oil source and peroxidation status interactively affect growth performance and oxidative status in broilers from 4 to 25 d of age. Poult. Sci..

[50] Viveros A (2009). Interaction of dietary high-oleic-acid sunflower hulls and different fat sources in broiler chickens. Poult. Sci..

[51] Zhang ZF, Zhou TX, Kim IH (2013). Effects of dietary olive oil on growth performance, carcass parameters, serum characteristics, and fatty acid composition of breast and drumstick meat in broilers. Asian-Australas. J. Animal Sci..

[52] Long S, Liu S, Wu D, Mahfuz S, Piao X (2020). Effects of dietary fatty acids from different sources on growth performance, meat quality, muscle fatty acid deposition, and antioxidant capacity in broilers. Animals.

[53] Zaki EF, El-Faham AI, Mohmed NG (2018). Fatty acids profile and quality characteristics of broiler chicken meat fed different dietary oil sources with some additives. Int. J. Health Animal Sci. Food Safety..

[54] Panda AK (2016). Effect of dietary incorporation of fish oil on performance, carcass characteristics, meat fatty acid profile and sensory attributes of meat in broiler chickens. Animal Nutr. Feed Technol..

[55] Ibrahim D, El-Sayed R, Khater SI, Said EN, El-Mandrawy SA (2018). Changing dietary n-6: n-3 ratio using different oil sources affects performance, behavior, cytokines mRNA expression and meat fatty acid profile of broiler chickens. Animal Nutr..

[56] Ebeid T, Fayoud A, El-Soud SA, Eid Y, El-Habbak M (2011). The effect of omega-3 enriched meat production on lipid peroxidation, antioxidative status, immune response and tibia bone characteristics in Japanese quail. Czech J. Animal Sci..

[57] Pekel AY (2012). Comparison of broiler meat quality when fed diets supplemented with neutralized sunflower soapstock or soybean oil. Poult. Sci..

[58] Omojola AB, Otunla TA, Olusola OO, Adebiyi OA, Ologhobo AD (2014). Performance and carcass characteristics of broiler chicken fed soybean and sesame/soybean based diets supplemented with or without microbial phytase. Am. J. Exper. Agric..

[59] Attia YA, Hassan SS (2017). Broiler tolerance to heat stress at various dietary protein/energy levels. Europ Poult Sci.

[60] Abdulla NR (2015). Fatty acid profile, cholesterol and oxidative status in broiler chicken breast muscle fed different dietary oil sources and calcium levels. South Afric. J. Animal Sci..

[61] Tufarelli V, Laudadio V, Casalino E (2016). An extra-virgin olive oil rich in polyphenolic compounds has antioxidant effects in meat-type broiler chickens. Environ. Sci. Pollut. Res..

[62] NRC. Nutrient Requirement of Poultry. National Academy of Sciences. 9th ed. Washington, DC: National Research Council (1994).

[63] AOAC. Official Methods of Analysis. Washington, DC: Association of Official Analytical Chemists (2004).

[64] Sklan D, Halevy O (1985). Digestion and absorption of protein along ovine gastrointestinal tract. J. Dairy Sci..

[65] Pinchasov Y, Nir I, Nitsan Z (1990). Metabolic and anatomical adaptations of heavy-bodied chicks to intermittent feeding. 2. Pancreatic digestive enzymes. Br. Poult. Sci..

[66] Sklan D, Hurwitz S, Budowski P, Ascarelli I (1975). Fat digestion and absorption in chicks fed raw or heated soybean meal. J. Nutr..

[67] Folch J, Lees M, Sloane-Stanley GH (1957). A simple method for the isolation and purification of total lipids from animal tissues. J Biol Chem.

[68] Peisker R (1964). Über das gemeinsame Vorkommen von angeborenen (Forbusschen) Aneurysmen und Anomalien im Bereich des basalen Gefäßringes. Acta Neurochir..

[69] Pearson, M.E Composition and Analysis of Foods. Ed. Ronald, S. Kirk and Ronald Sawyer, Ninth Edition. 640–642 (1991).

[70] Hood DE (1980). Factors affecting the rate of metmyoglobin accumulation in prepackaged beef. Meat Sci..

[71] Meek KI, Claus JR, Duncan SE, Marriott NG, Solomon MB, Kathman SJ, Marini ME (2000). Quality and sensory characteristics of selected post-rigor, early deboned broiler breast meat tenderized using hydrodynamic shock waves. Poult. Sci..

[72] Commission Internationale de l’Éclairage (CIE). Recommendations on Uniform Colour Spaces, Colour Difference Equations, Psychometrics Colour Terms, Supplement No. 2 to Publication CIE No. **715** (E1–131) (1978).

[73] SAS, Institute. SAS R User’s Guide for Personal Computer. Cary, NC: SAS Institute Inc. (2004).

